# Virtual Reality for Neurorehabilitation and Cognitive Enhancement

**DOI:** 10.3390/brainsci11020221

**Published:** 2021-02-11

**Authors:** Danko D. Georgiev, Iva Georgieva, Zhengya Gong, Vijayakumar Nanjappan, Georgi V. Georgiev

**Affiliations:** 1 Institute for Advanced Study, 9010 Varna, Bulgaria; ivavgeorgieva@gmail.com; 2 Center for Ubiquitous Computing, University of Oulu, FI-90014 Oulu, Finland; zhengya.gong@oulu.fi (Z.G.); vijayakumar.nanjappan@oulu.fi (V.N.); georgi.georgiev@oulu.fi (G.V.G.)

**Keywords:** brain cortex, cognition, motor control, neurorehabilitation, perception, robotic devices, self-enhancement, virtual reality

## Abstract

Our access to computer-generated worlds changes the way we feel, how we think, and how we solve problems. In this review, we explore the utility of different types of virtual reality, immersive or non-immersive, for providing controllable, safe environments that enable individual training, neurorehabilitation, or even replacement of lost functions. The neurobiological effects of virtual reality on neuronal plasticity have been shown to result in increased cortical gray matter volumes, higher concentration of electroencephalographic beta-waves, and enhanced cognitive performance. Clinical application of virtual reality is aided by innovative brain–computer interfaces, which allow direct tapping into the electric activity generated by different brain cortical areas for precise voluntary control of connected robotic devices. Virtual reality is also valuable to healthy individuals as a narrative medium for redesigning their individual stories in an integrative process of self-improvement and personal development. Future upgrades of virtual reality-based technologies promise to help humans transcend the limitations of their biological bodies and augment their capacity to mold physical reality to better meet the needs of a globalized world.

## 1. Introduction

The rapid development of digital technologies has transformed societies across the world [1,2]. Access to electronic devices and the internet exposes our minds to virtual computer-generated worlds, which greatly impact our daily lives [3,4,5]. If exposure to virtual realities is subordinate to achieving long-term personal goals, digital technologies are able to improve the overall well-being of healthy individuals [6,7,8]. Furthermore, technologies employing virtual realities may be helpful to older adults suffering from cognitive decline and social isolation [9], may assist neurorehabilitation of patients with stroke [10] or traumatic brain injury [11], and may even be an essential ingredient for the replacement of lost functions through an appropriate brain–computer interface (BCI) that controls robotic devices [12,13,14,15,16].

The interaction between the human mind and virtual realities has been demonstrated to improve cognitive functions [17,18,19,20,21,22]. Biologically, this effect cannot be achieved without the activation of some forms of neural plasticity, such as strengthening or attenuation of synaptic transmission [23], remodeling of synaptic connections [24], reshaping of dendritic spines [25,26,27,28], reorganization of neuronal morphology [29,30,31], or modulation of electric excitability [32,33,34]. Direct evidence for the underlying molecular changes at the level of individual neurons, however, is beyond the reach of current methods for functional brain imaging. Nevertheless, electroencephalography (EEG) [35,36], magnetoencephalography (MEG) [37,38], near-infrared spectroscopy (NIRS) [39,40], positron emission tomography (PET) [41,42,43], and magnetic resonance imaging (MRI) [44,45,46] can resolve functional brain states with macroscopic resolution (e.g., EEG has a temporal resolution of milliseconds and MRI has a spatial resolution of millimeters) that can detect cumulative changes in brain volume or excitability acquired over several weeks of training or rehabilitation.

In this present review, we will first portray different types of virtual reality (VR) employed in biomedical practice and will concisely describe their measurable impact upon brain structure and cognitive performance. Then, we will explore important medical applications of VR technologies that significantly improve the quality of life in patients with neurological deficits. Lastly, we will conclude with the promises VR use offers healthy individuals for self-improvement and personal development.

## 2. Types of Virtual Reality

Computer-generated worlds provide digital experiences, which are referred to as virtual realities. Depending on the intensity and quality of feelings elicited by the computer-generated world, several main types of virtual realities can be differentiated.

### 2.1. Non-Immersive Virtual Reality

In this type of reality, the person is not fully immersed in the virtual world [47]. It is the most common type of VR encountered by us while working with personal computers, tablets, smartphones, television sets, or other electronic devices. Because the virtual world is displayed on computer monitors or large television screens, and the interaction happens through input devices like keyboards, mice, or controllers, the person does not have the feeling of being present inside the virtual world. Instead, the person may experience simultaneously both the real world, e.g., the physical surroundings in the room, and the contents of the virtual world, e.g., the position of an avatar inside a computer game.

### 2.2. Fully Immersive Virtual Reality

In this type of reality, the person is fully immersed and has the feeling of presence in the virtual world [48,49]. The person enters into the virtual world with the help of specialized hardware, such as a head-mounted display (HMD), a bodysuit, data gloves, and an immersive room. The purpose of this extra equipment is to eliminate the sensory flow of information from the real world [50] and substitute it with the computer-generated one. This sustains the illusion experienced by the person that the virtual world is the actual real world. Sensors attached to the bodysuit can be used to monitor the person’s movements, and an EEG cap can be used to track brain activity. Thus, the act of immersion is accompanied by the generation and recording of large amounts of experimental data, which can be collected and analyzed in a retrospective fashion.

### 2.3. Augmented Reality

A characteristic feature of augmented reality is that some components of the virtual world are superimposed on the surrounding world [51,52]. The person experiences computer-generated perceptual information that is overlaid on physical objects residing in the real-world environment. Electronic devices equipped with cameras, such as smartphones and tablets, currently allow for capturing snapshots of the real world that can be enhanced with animations or other digital information selected from VR applications. A practical way for augmenting reality is through the visual system using hands-free wearables, such as smart glasses. In augmented reality, the user can see the components of the virtual world but is not able to interact with them.

### 2.4. Mixed Reality

This type of hybrid reality is a form of augmented reality in which the real elements and the virtual elements are able to interact with one another, thereby granting the user the ability to interact with both real and virtual objects [53,54,55]. Further development of digital technologies may even allow for the projection of three-dimensional holograms in real space and user interaction with projected digital controllers as needed.

### 2.5. Extended Reality

Extended reality (XR) is a general term that encompasses all immersive technologies, including present-day technologies, such as the aforementioned augmented reality (AR), VR, or mixed reality (MR), plus future technologies that are still to be created. The application of such advanced technologies in the context of health emergencies deserves further consideration as this will create opportunities for effective non-human interaction.

## 3. Cortical Localization of Cognitive Functions

The seat of human consciousness is located in the brain cortex, which forms the outer layer of the cerebrum [56,57,58]. In large mammals and primates, the brain cortex is folded into grooves (sulci) and ridges (gyri), which are tightly packed within the limited space available inside the skull [59,60]. Although different higher cognitive functions seem to be flawlessly integrated into a single stream of conscious experience [61,62], different parts of the brain cortex have been shown to play different specialized roles, as evidenced by localized cerebral lesions [63,64]. This localization of cognitive functions in the brain cortex has been further corroborated by modern techniques for functional brain imaging [65,66] and can be exploited by VR technologies that rely on BCIs [67,68].

Knowledge of the cortical anatomy is essential for the accurate description of the localization of cognitive functions and proper understanding of the localized nature of observed changes in gray matter volumes or EEG power spectra after VR exposure. With an interdisciplinary audience of biomedical engineers, computer scientists, health professionals, and neuroscientists in mind, we briefly outline the characteristic anatomical features of the human brain cortex and summarize their relevance to cognition.

Structurally, the cerebrum consists of two cerebral hemispheres, designated as left and right respectively. Each hemisphere has an outer layer of gray matter, referred to as the cortex, and an inner layer of white matter. The cortex is further divided by large grooves into four lobes: frontal lobe, temporal lobe, parietal lobe, and occipital lobe. Each lobe contains ridges, referred to as gyri, specialized in the execution of specific cognitive functions.

### 3.1. Frontal Lobe

**Figure 1:**
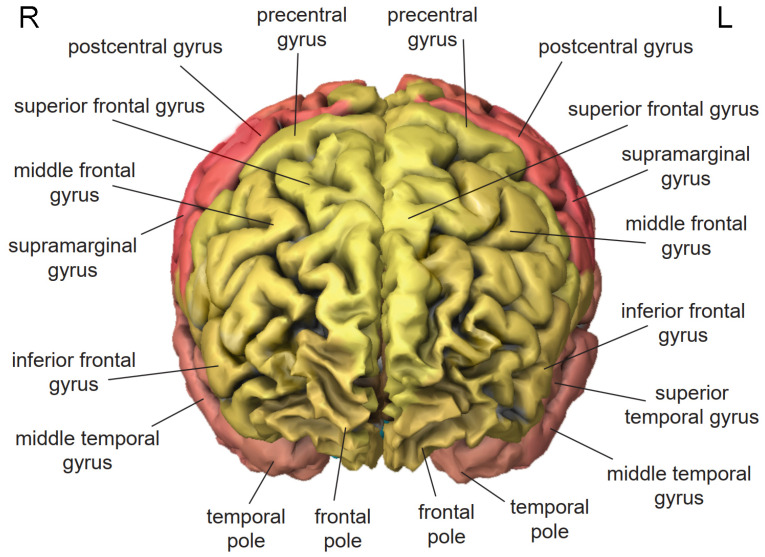
Frontal view of the human brain based on H0351.2002 dataset in Allen Brain Atlas. Frontal lobe (yellow), parietal lobe (red), temporal lobe (pink). L, left; R, right.

**Figure 2:**
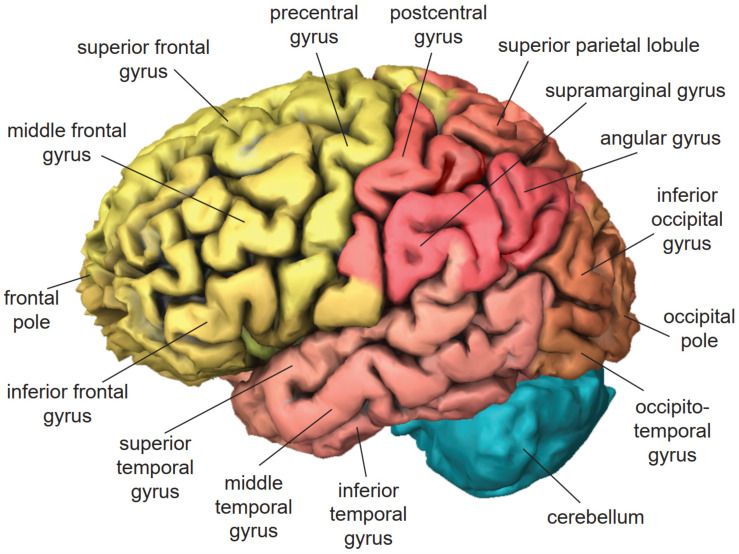
Lateral view of the left hemisphere of the human brain based on H0351.2002 dataset in Allen Brain Atlas. Frontal lobe (yellow), parietal lobe (red), temporal lobe (pink), occipital lobe (salmon), cerebellum (turquoise).

**Figure 3:**
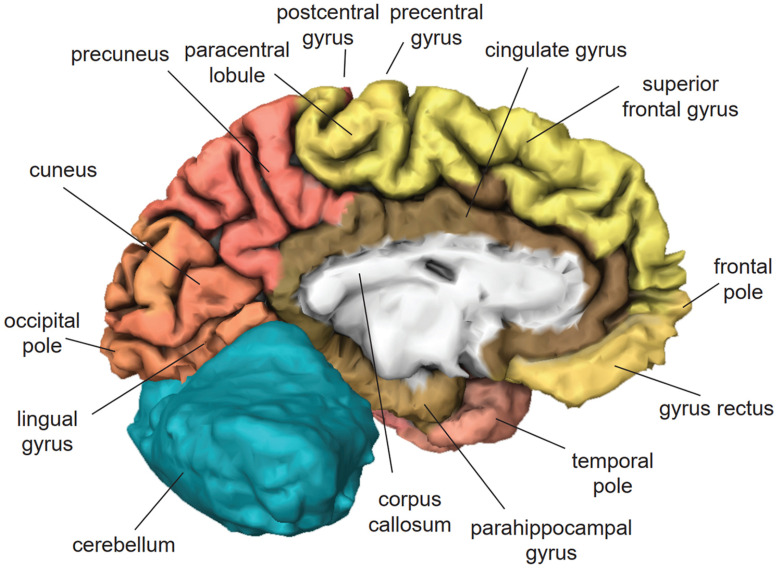
Midsagittal view of the left hemisphere of the human brain based on H0351.2002 dataset in Allen Brain Atlas. Frontal lobe (yellow), parietal lobe (red), temporal lobe (pink), occipital lobe (salmon), limbic system (brown), corpus callosum (white), cerebellum (turquoise).

The frontal lobe is located at the front of the head [56] (Figure 1). In VR applications, it is actively involved in working memory and motor control [69,70]. The precentral gyrus contains the primary motor cortex, which exercises control over voluntary movement through stimulating contraction of skeletal muscles. The superior frontal gyrus is implicated in self-awareness [71] and the generation of laughter [72]. The middle frontal gyrus (Figure 2) exerts control over automatic behavior [73], contributes to maintaining information in consciousness, and is recruited primarily when information must be manipulated in working memory [74,75,76]. The inferior frontal gyrus of the dominant hemisphere contains Broca’s area, which controls the production of speech and expressive language [77]. The cingulate gyrus (Figure 3) is involved in sensory perception of pain induced by noxious stimuli [78], the encoding of negative memories [79], and avoidance learning for physical events that are associated with negative outcomes [80].

### 3.2. Temporal Lobe

The temporal lobe is located on the side of the head above the ear [56] (Figure 2). In VR applications, it is actively involved in the semantic processing of information and episodic memory [81,82]. The superior temporal gyrus of the dominant hemisphere contains Wernicke’s area, which is essential for the understanding of written and spoken language [83]. The middle temporal gyrus is involved in sound recognition, semantic retrieval, semantic memory, and language processing [84]. The inferior temporal gyrus contributes to the execution of word-retrieval tasks [85]. The fusiform gyrus contributes to the processing of color information and face recognition [86]. The parahippocampal gyrus (Figure 3) is responsible for the encoding and retrieving of memories [87].

### 3.3. Parietal Lobe

**Figure 4:**
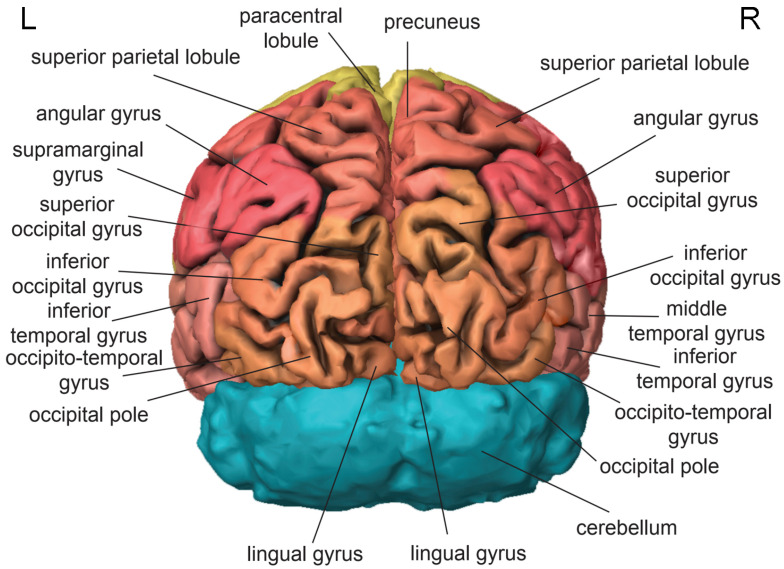
Posterior view of the human brain based on H0351.2002 dataset in Allen Brain Atlas. Frontal lobe (yellow), parietal lobe (red), temporal lobe (pink), occipital lobe (salmon), cerebellum (turquoise). L, left; R, right.

The parietal lobe is located in the middle-upper part of the head above the temporal lobe [56] (Figure 2). In VR applications, it is actively involved in creating the feeling of presence [88,89,90,91]. The postcentral gyrus contains the primary somatosensory cortex, which generates somatic sensations and the feeling of embodiment [92]. The superior parietal lobule (Figure 4) is involved in visual imagery [93], mental transformations of the body-in-space [94], and regulation of emotions [95]. The supramarginal gyrus contributes to proprioception [96], emotional responses [95], and the phonological processing of spoken and written language [97,98]. The angular gyrus plays a role in mental calculation [99], the encoding and retrieval of schema-associated memories [100], and imagination [101]. The precuneus (Figure 3) contributes to visuospatial imagery, retrieval of episodic memories, and self-processing operations, such as taking a first-person perspective or experiencing agency [102].

### 3.4. Occipital Lobe

The occipital lobe is located at the back of the head [56] (Figure 4). In VR applications, it is actively involved in creating visual images [103,104]. The primary visual cortex, which is responsible for vision, is mostly buried in the calcarine fissure located on the medial surface of the occipital lobe [105,106,107], but it also extends in the cuneus and the lingual gyrus, which flank the calcarine fissure on the top and bottom, respectively. The cuneus is involved in the basic processing of visual information received from the retina [108,109]. The lingual gyrus plays an important role in the process of reading, namely, the identification and recognition of words [110]. The superior, middle, and inferior occipital gyri contain visual association cortices, which interpret and give additional meaning to visual signals [111].

## 4. Virtual Reality for Neurorehabilitation

Brain injury is a serious medical condition that disrupts the normal functioning of the brain and severely impacts a person’s life. Two major causes of brain damage are mechanical trauma, which is the most common type of brain injury seen in younger adults (<45 yo) [112], and vascular incidents (stroke), more commonly seen in older adults (>45 yo) [113]. Traumatic brain injury (TBI) and stroke lead to cognitive, neurological, and psychological disabilities that can be partially recovered by neurorehabilitation [114]. The most common types of disability resulting from brain injury are: paralysis or impaired motor control; sensory disturbances, including pain; cognitive disturbances, including compromised understanding or language use (aphasia), and impaired thinking and memory; and emotional disturbances, including feelings of fear, anxiety, frustration, or sadness. Inclusion of VR in the rehabilitation process has shown a promise for better functional outcomes, including the recovery of the damaged neural tissue and compensation of any functional alterations resulting from the injury [115].

### 4.1. Motor Rehabilitation

VR provides a safe, controlled environment for performing customizable, engaging rehabilitation activities that promote learning of motor skills [116]. Furthermore, because VR is fun and enjoyable, it motivates children to participate in the rehabilitation interventions [117]. The therapeutic effect of VR can be easily combined with computer-assisted cinematic analysis of motor deficits after brain lesions [118]. This allows for a reliable documentation of the degree of motor impairment in brain-injured patients undergoing rehabilitation therapy. Because the virtual environments are highly interactive, they strongly activate visual, vestibular, and proprioceptive systems during the execution of a virtual task, such as playing a video game. Immersion into the game can be achieved using head-mounted displays [119], which are accessible for all segments of the population at a relatively low cost and can be used for rehabilitation even in a typical home environment [120].

The main therapeutic effect of VR on upper limb motor activity is to increase the active range of motion (AROM) of the shoulder, elbow, and wrist [121,122]. Significant gray matter increases were detected by MRI with voxel-based morphometry in five brain areas: the tail of the hippocampus, the left caudate nucleus, the rostral cingulate zone, the depth of the central sulcus, and the visual cortex [122]. Furthermore, the gray matter volumes of motor, premotor, and supplementary motor cortices correlated positively with the power and AROM measured in motor tests [122]. Interestingly, EEG recordings showed significantly increased EEG concentration (indicated by strong beta waves) in the frontopolar 2 (FP2) and frontal 4 (F4) areas, and enhanced brain activity (indicated by higher average wave frequency) in the frontopolar 1 (FP1) and frontal 3 (F3) areas in an upper-extremity training group using VR [123]. The most important feature of VR interventions, however, is that the improved upper limb motor function recovers activities of daily living (ADL) of brain-injured patients and enhances their quality of life [124].

Brain injuries that affect motor cortex areas innervating the lower limb may result in impairments in the gait, maintenance and adaptation of balance, or postural control for a range of activities of daily living [125]. Because the working load on the lower limbs during walking also includes support of the person’s body weight, gait rehabilitation is greatly assisted by robotic devices, which allow a smaller workforce and a longer exercise session with greater intensity compared to traditional treatment [126]. Lokomat is one such robotic device equipped with electronic control that allows connection to a non-immersive VR screen on which an avatar delivers visual feedback of the patient’s movements. Inclusion of the VR feedback was found to significantly improve the patient’s mood, perception of physical well-being, global cognitive functions, executive functions (such as perseveration, planning, and classification), cognitive flexibility, and selective attention—all of which impacted positively on the patient’s quality of life [126]. Gait and balance interventions may also include a moving platform with an integrated treadmill that participants use to interact with a virtual environment. Projection of synchronized VR environments on a 180 degree cylindrical screen allows subjects to walk around and move in an attractive and engaging environment, which is particularly beneficial for the rehabilitation of children [127]. Similar to upper limb rehabilitation, the act of learning to control a walking avatar in the absence or presence of visuomotor perturbations lead to observable cortical adaptations in EEG activities [128], which indicates underlying neural plasticity and neural reorganization.

### 4.2. Cognitive Rehabilitation

The use of VR allows for a reproducible, objective assessment of cognitive processes underlying attention, memory, information processing, logical sequencing, and problem-solving [47,129]. VR also provides a safe environment in which to assess skills that might be too dangerous or risky to perform in the real world (e.g., cooking or driving), and the tested subjects are able to make mistakes without suffering the real consequences [129,130,131]. The stimulating effect of VR on the human mind is highly beneficial for cognitive rehabilitation. Brain injuries often display impairments of attention, memory, affectivity, behavior, planning, or executive functions [132]. Prospective memory failure, which is manifested as an inability to recall delayed intentions, is a serious problem that hinders everyday activities and heavily burdens the patients that experience it [133]. Non-immersive VR-based cognitive rehabilitation programs that run on a desktop computer allows for cost-effective training of patients [134] by enabling them to practice prospective memory tasks, such as preparing coffee in a virtual kitchen [131,135], operating an automated teller machine (ATM) to access their bank accounts [136], or purchasing items from a shopping list in a virtual convenience store [133,137]. Such VR-based training is well accepted by the patients and has demonstrated encouraging improvement in cognitive attributes that depend on frontal lobe functions, including immediate recall of prospective memory tasks and accurate execution of event-based, time-based, and ongoing tasks [138]. Significant improvements in learning following a VR exercise program are thought to be associated with changes in neuronal plasticity that enhance the working memory [139]. VR also significantly increases cognitive flexibility, shifting skills, and selective attention, leading to better behavioral outcomes in brain-injured patients [140]. Improvement in selective memory processes and problem-solving skills facilitate social reintegration and leads to better vocational outcomes [141].

### 4.3. Emotional Rehabilitation

Brain injury often leads to anxiety, depression, emotional lability, and mood swings. Medication with antidepressants [142] or mood stabilizers [143] could be potentiated by emotional rehabilitation [144,145] that helps the patient overcome the pain of loss and return to a more stable, healthier place. Training in VR utilizes the positive effects of environmental enrichment [146] to trigger the neural mechanisms of recovery, including hippocampal neuroplasticity and neurogenesis [147,148], which have been implicated in the stress response and control of emotions [149], and are essential for the behavioral effects of antidepressants [150,151,152]. VR could also be used as a novel engagement tool that helps patients to understand their condition better, thereby increasing the reported level of understanding, comfort, and satisfaction [153]. The use of immersive VR further allows the combining of experiential enrichment and physical exercise, which greatly improves social, psychological, and emotional health [154]. The beneficial effects of exercise originate from structural and neurochemical adaptations in the central nervous system [155], including changes in several neurotransmitter systems, such as increased levels of catecholamines [156,157,158,159], which in turn increase attention, sharpen focus on performed tasks, enhance memory storage, and induce feelings of happiness [160].

### 4.4. Sensory Rehabilitation

Sensory deficits, including pain, may persist as long-term symptoms of traumatic injuries. In such cases, immersive VR could be used as a form of distraction analgesia alone or in combination with a pharmacological intervention (such as opioid administration) [161]. Maladaptive plasticity of the primary sensorimotor cortex, following deprivation of sensory input due to limb amputation, may lead to phantom pain, the management of which can be challenging [162]. One therapeutic method with proven efficacy for patients with post-amputation pain is the extended viewing in a mirror box of the movements performed by their intact limb [163,164]. Recent developments in immersive VR technologies allow the implementation of a VR mirror box, which was found to activate the primary sensorimotor cortex much more potently than the classical mirror box condition [165]. Thus, VR can build upon and improve the efficacy of conventional methods for pain management.

## 5. Virtual Reality for Replacement of Function

Severe brain injuries, which cause irreversible damage to neural tissue, may result in permanent loss of motor or sensory function. However, because the seat of human consciousness is located in the brain cortex, it is possible to replace lost functions with the use of BCIs, provided that the damage involves only the peripheral nervous system or the peripheral effector organs. In other words, the brain cortex can be directly connected to bionic devices, which are engineered to perform the lost functions of the damaged peripheral organs.

### 5.1. Replacement of Motor Function

Severe paralysis may be caused by different pathogenetic mechanisms, such as spinal cord trauma [166], neurodegenerative diseases affecting the motor neurons [167], autoimmune diseases causing muscle weakness [168], or genetic muscular dystrophies [169]. For severely paralyzed people, the use of a BCI permits successful re-establishment of communication with the surrounding world [170,171]. Because the muscles of paralyzed patients undergo disuse atrophy [172], the replacement of motor function is usually achieved through control of robotic devices. Surgically implanted BCIs detect electric signals from the cortical surface using electrocorticography (ECoG), which ensures high spatial resolution [173]. For reliable control of external robotic devices, however, the electric activity should be recorded from regions of the brain cortex where voluntary mental operations could elicit certain discernible wavefronts, such as sensorimotor rhythms (SMRs) or the so-called P300 evoked response. SMR signals recorded over the sensorimotor cortex can be elicited voluntarily through motor imagination [67,68,174]. For example, during the act of imagined opening/closing of the hand, an event-related synchronization/desynchronization can be recorded on the ipsilateral/contralateral cortex in the EEG frequency band of 8–13 Hz [175]. The P300 response recorded from the parietal lobe [176,177] is an event-related potential component, which is elicited in the process of decision-making [178]. Because it is quite difficult to control one’s own EEG signals, training protocols need to provide visual feedback that allows the subjects to monitor their progress [179]. VR can provide such feedback for tracking the progress of a BCI-controlling task [180] and can even sustain the illusion of embodiment through suitable sensory stimulation to reward specific brain-activity patterns [181,182,183,184,185,186]. Indeed, transcranial magnetic stimulation (TMS) has been successfully applied to achieve a sense of ownership and a sense of agency over an avatar in immersive VR [187]. The substitution of one’s own body with a virtual body results in corresponding changes in perception, attitude, and behavior [188]. While the experimental swapping of bodies [189,190,191,192] and the body ownership illusion [188,193] may be considered as recreational applications in healthy individuals, the sense of embodiment provides disabled individuals with much more precise and reliable control over BCI-connected robotic devices. Thus, with the advent of VR technologies, it is possible to embody paralyzed individuals and endow them with BCI control over robotic devices, such as robotic arms, spellers, wheelchairs, or drones, all embedded with sensors and running specialized software for the purpose of connecting and exchanging data with other devices or systems over the internet [175]. Replacement of lost functions through BCIs in paralyzed or locked-in patients [15,194,195] gives them the chance of having a meaningful, dignified life.

### 5.2. Replacement of Sensory Function

Direct electric stimulation of the brain cortex is able to elicit conscious experiences in awake subjects (e.g., during neurosurgery) [196]. This fact could be exploited for the restoration of vision in blind patients through BCIs implanted in the visual cortex [197,198]. Traumatic injury of the eyes and their retinas leads to blindness due to malfunction of the peripheral sensory transduction of incoming light images into a series of electric spikes. For a functional replacement of the retina, bionic devices consisting of a charge-coupled device (CCD) digital camera connected to a portable computer, which processes the image in order to detect edges and perform black/white reversal, could be used. The processed image can then be delivered through electric stimulation of the visual cortex to produce phosphenes, which are colorless flashes of light on a black background [198,199,200,201]. Through the experience of phosphenes, a patient with bionic vision was capable of accomplishing a complex task, such as walking across a room, pulling a ski hat off a wall, and correctly putting the hat on the head of a mannequin [198,202]. The same patient also demonstrated that the bionic vision is useful for navigation in unfamiliar environments as he was able to ride the subway of a large city [198]. Thus, the bionic restoration of senses greatly improves the quality of life and facilitates social integration.

## 6. Virtual Reality for Self-Enhancement

VR shapes modern life, including entertainment and digital health. As with every tool, the quality of its use depends on the intentions of the user. Augmented reality provides easy access to vast amounts of computer-stored data, which is an ideal way to enhance users’ creative problem-solving and decision-making [203,204,205,206]. VR could easily simulate any specific physical environment, such as a mountain [207], a forest [208], a beach [209], or a savannah [210], which could evoke positive emotions and hence improve cognitive abilities. Because VR creates a storytelling experience, it is also able to profoundly affect the way we view ourselves and the surrounding world. This provides us with an opportunity to arrange individual life events into a story, which unfolds in settings that are designed to aid the self (for coping with frustration or resolving psychological conflicts). The presence of challenging experiences may profoundly change the way in which individuals perceive their life narratives and store their memories. Immersion in VR enables exploration of alternative scenarios that supply a vision of one’s overall life trajectory in a more sensible and healthier way [211,212]. Taking into consideration the importance of the narrative self for discovering an individual’s purpose in life, VR immersion could be utilized as a medium for the construction of a new storyline with a different attitude toward the past [7,8]. This approach will also revisit the attitudes toward the present moment and the future, and thus will better shape the narrative of the self for achieving healthier life experiences [213,214]. Thus, VR technology is ideally suited to aid self-improvement, which is about ending negative behaviors, and promote personal-development, which is about learning, growing, expanding awareness, and developing one’s full potential. Maintaining a healthy state of mind and body facilitated by VR experiences allows one to live an exciting life in which one can take one’s dreams and aspirations to the next level.

## 7. Conclusions

VR presents a breakthrough in the capability of technology to recreate reality and so it embodies the philosophical concept of the virtual [215] into a practical mode of experience. The concept of the virtual originates from the ontological concept of the illusion of reality [216]. Present-day VR, however, is a brilliant new medium that exceeds illusion and brings about tangible results in reality with unlimited potential for large-scale application in art, entertainment, relaxation, learning, exercise, training, and treatment or therapy. VR visualizes not only events but also psychological conditions and personalized perceptions [217,218,219,220], induces a sense of ownership [221,222] and of presence [223,224,225,226,227], offers immersion [228], and renders the self in different reality modes, such as being represented by avatar or having a different gender [229,230]. It also influences physical sensations in interventions, such as pain management [231,232] or stress and anxiety reduction [233]; induces necessary emotions, such as empathy [234]; or aims at achieving higher goals, such as self-development [235].

VR promises a plethora of experiences to people who engage in it and induces states of mind ranging from simplified to overwhelming. The scale of these states goes from pure excitement or fear [236,237] to more sophisticated ones that are combined with body states induced by exercise or meditation, such as training and learning new skills [238], deep relaxation, and general support of well-being [239,240]. VR may also help healthy individuals to redesign themselves in view of achieving a much more meaningful, purposeful, and exciting life.

Contemporary use of VR goes far beyond entertainment. It can be beneficial for training, for research purposes, and for neurorehabilitation. BCIs may assist the replacement of lost functions, such as moving or speaking, thereby restoring severely paralyzed or locked-in patients’ ability to communicate with the surrounding world. VR may additionally support the perception of an embodiment for precise control over bionic devices that extend the capabilities of the human body.

## Data Availability

H0351.2002 dataset used for rendering images of the human brain is publicly available from Allen Brain Atlas (https://www.brain-map.org). All brain reconstructions were rendered with Brain Explorer 2.3.5 (https://human.brain-map.org/static/brainexplorer), which can be freely downloaded and installed on Windows or Mac Operating Systems.

## Abbreviations

The following abbreviations are used in this manuscript:

ADL	activities of daily living
AR	augmented reality
AROM	active range of motion
ATM	automated teller machine
BCI	brain–computer interface
CCD	charge-coupled device
ECoG	electrocorticography
EEG	electroencephalography
HMD	head-mounted display
MEG	magnetoencephalography
MR	mixed reality
MRI	magnetic resonance imaging
NIRS	near-infrared spectroscopy
PET	positron emission tomography
SMR	sensorimotor rhythm
TBI	traumatic brain injury
TMS	transcranial magnetic stimulation
VR	virtual reality
XR	extended reality
